# Dynamics of ventilatory pattern variability and Cardioventilatory Coupling during systemic inflammation in rats

**DOI:** 10.3389/fnetp.2023.1038531

**Published:** 2023-07-31

**Authors:** Cara K. Campanaro, David E. Nethery, Fei Guo, Farhad Kaffashi, Kenneth A. Loparo, Frank J. Jacono, Thomas E. Dick, Yee-Hsee Hsieh

**Affiliations:** ^1^ Division of Pulmonary, Critical Care and Sleep Medicine, Department of Medicine, Case Western Reserve University, Cleveland, OH, United States; ^2^ Institute for Smart, Secure and Connected Systems (ISSACS), Case Western Reserve University, Cleveland, OH, United States; ^3^ Division of Pulmonary, Critical Care and Sleep Medicine, Department of Medicine, Louis Stokes Cleveland VA Medical Center, Cleveland, OH, United States; ^4^ Department of Neurosciences, Case Western Reserve University, Cleveland, OH, United States

**Keywords:** cardiorespiratory coupling, heart rate variability, ventilatory pattern variability, plethysmography, and telemetry

## Abstract

**Introduction:** Biometrics of common physiologic signals can reflect health status. We have developed analytics to measure the predictability of ventilatory pattern variability (VPV, Nonlinear Complexity Index (NLCI) that quantifies the predictability of a continuous waveform associated with inhalation and exhalation) and the cardioventilatory coupling (CVC, the tendency of the last heartbeat in expiration to occur at preferred latency before the next inspiration). We hypothesized that measures of VPV and CVC are sensitive to the development of endotoxemia, which evoke neuroinflammation.

**Methods:** We implanted Sprague Dawley male rats with BP transducers to monitor arterial blood pressure (BP) and recorded ventilatory waveforms and BP simultaneously using whole-body plethysmography in conjunction with BP transducer receivers. After baseline (BSLN) recordings, we injected lipopolysaccharide (LPS, *n* = 8) or phosphate buffered saline (PBS, *n* =3) intraperitoneally on 3 consecutive days. We recorded for 4–6 h after the injection, chose 3 epochs from each hour and analyzed VPV and CVC as well as heart rate variability (HRV).

**Results:** First, the responses to sepsis varied across rats, but within rats the repeated measures of NLCI, CVC, as well as respiratory frequency (fR), HR, BP and HRV had a low coefficient of variation, (<0.2) at each time point. Second, HR, fR, and NLCI increased from BSLN on Days 1–3; whereas CVC decreased on Days 2 and 3. In contrast, changes in BP and the relative low-(LF) and high-frequency (HF) of HRV were not significant. The coefficient of variation decreased from BSLN to Day 3, except for CVC. Interestingly, NLCI increased before fR in LPS-treated rats. Finally, we histologically confirmed lung injury, systemic inflammation via ELISA and the presence of the proinflammatory cytokine, IL-1β, with immunohistochemistry in the ponto-medullary respiratory nuclei.

**Discussion:** Our findings support that NLCI reflects changes in the rat’s health induced by systemic injection of LPS and reflected in increases in HR and fR. CVC decreased over the course to the experiment. We conclude that NLCI reflected the increase in predictability of the ventilatory waveform and (together with our previous work) may reflect action of inflammatory cytokines on the network generating respiration.

## 1 Introduction

Sepsis results from a dysregulated host response to infection and causes life-threatening organ dysfunction (Singer et al., 2016). Early diagnosis of sepsis is a key factor to improving survival (Liu et al., 2017). Unfortunately, the most definitive diagnosis depends on growing microbial cultures, which requires time and delays treatment (Liu et al., 2017). Scoring systems based on physiologic biometrics can predict a patient’s prognosis but do not assist diagnosis (e.g., qSOFA, NEWS) (Seymour et al., 2016; Shankar-Hari et al., 2016; Singer et al., 2016). Given the benefit of early detection of sepsis, we (and others) have sought alternative biometrics to aid in early identification of sepsis (Vandendriessche et al., 2014; Buchman et al., 2020; Monfredi et al., 2021). We have focused on ventilatory pattern variability (VPV) and developed the Nonlinear Complexity Index (NLCI) that quantifies the predictability of a continuous waveform associated with inhalation and exhalation. Values of NLCI increase early in the development of sepsis and acute lung injury (Jacono and Dick, 2011; Young et al., 2019; Hsieh et al., 2020). However, an effective biometric would be stable during a steady state but be sensitive to state changes and thus track a patient’s disease trajectory and response to treatment (Talisa et al., 2018).

We define the states of health and disease by a set of values of physiologic variables. Ideally, these physiologic variables are measured easily and reliably for a given state and differ from healthy and disease states. Much research has focused on heart rate and especially heart rate variability (HRV) (Vandendriessche et al., 2017a). We theorized that additional biometrics related to breathing would complement HRV and provide a more robust set of physiologic variables. Indeed, breathing pattern variability outperformed HRV in predicting success of weaning patients from a ventilator (Seely et al., 2014). Ideally, we want to sample stationary data; thus, we anticipated low variability in measures of a given period, e.g., clustered epochs from the same 30-min period and then more significant differences across the days of the experimental protocol. Thus, a goal of this study was to measure NLCI and other indexes of Cardiorespiratory Coupling from a healthy state to a developing systemic inflammatory state.

The quintessential example of a reliable physiologic variable is heart rate (HR). HR has beat-to-beat variability in healthy individuals. In patients with systemic inflammation, HR increases due to decreased vagal nerve activity and increased epinephrine, while heart rate variability (HRV) decreases (Goldberger, 1999; Vandendriessche et al., 2014; Chiew et al., 2019). The decrease in HRV is evident in the frequency-domain with decreased high-frequency (HF) component of the power spectral density. The HF component of HRV depends on coupling between respiration and the cardiovascular systems (Shaffer and Ginsberg, 2017; Pham et al., 2021). Our previous work described that Cardiorespiratory Coupling (CRC) decreases during systemic inflammation, which may result from brainstem inflammation (Dick et al., 2014; Hsieh et al., 2020).

The development of nonlinear analyses to quantify biologic variability *complements* linear approaches. The combination of linear and nonlinear analytical tools provides greater value in defining the progression of systemic inflammation to sepsis (Monfredi et al., 2021). For HRV in a pre-clinical model of sepsis, multiscale sample entropy of beat-to-beat dynamics was a more sensitive biometric than linear indices of variability, unifractal dynamics, HR or blood pressure (BP) (Vandendriessche et al., 2014).

Cardioventilatory Coupling (CVC) refers to a weak relationship between the occurrence of a heartbeat and the onset of inspiration. Specifically, CVC is the tendency for the last heartbeat in expiration to occur at a preferred latency before inspiratory onset (Galletly and Larsen, 1998; Friedman et al., 2012; Barnett et al., 2021). CVC depends on baroreceptor sensory input, which terminates in the nucleus Tractus Solitarii (nTS) (McMullan et al., 2009). We have shown that the integration of sensory input in distributed network that generates the respiration is sensitive to neuro-inflammation (Litvin et al., 2018; Getsy et al., 2019). Thus, we hypothesize that CVC should be attenuated in LPS-treated rats.

The goal of our work is to determine the dynamics of the ventilatory waveform by quantifying the shape of the airflow signal throughout the respiratory cycle over time. We refer to these metrics as ventilatory pattern variability (VPV). Further, we have established a measure that quantifies nonlinear variability in the respiratory signal defined as NLCI (Dhingra et al., 2011). Previously, we reported that the NCLI had good specificity and selectivity in assessing increasing severity of lung injury caused by intratracheal installation of bleomycin (Young et al., 2019). After lung injury, we associated a greater predictability in VPV with the presence of pro-inflammatory cytokines in the brainstem (Jacono et al., 2011; Hsieh et al., 2020). Here, we test the hypothesis that a general systemic inflammatory response evoked by repeated injections of lipopolysaccharide (LPS) will lead to progressive changes in VPV, CVC, and HRV. We hypothesize that VPV and cardiorespiratory coupling better reflect changes health status during repeated injections of LPS and may be used to predict the inflammatory state.

## 2 Methods

The Case Western Reserve University Institutional Animal Care and Use Committee approved all surgical and experimental procedures and ensured that they adhered to the guidelines published by NIH National Research Council (US) Committee for Guidelines for the Care and Use of Laboratory Animals updated in 2011.

### 2.1 Experimental model (instrumentation of the rats)

We implanted a pressure transducer transmitter (PA-C40, Data Sciences International, Minneapolis, MN, United States) to measure blood pressure. First, we anesthetized rats (adult, male Sprague-Dawley rats (*n* = 11, 250–300 gm) by an intraperitoneal injection of a solution containing ketamine, xylazine, and acepromazine (100, 10 and 5 mg/kg bw; respectively). Then, we exposed the descending aorta and inserted the transducer’s catheter. The catheter was tied and sealed with adhesive to measure BP. Finally, the transducer was sutured to the abdominal wall and the incision site was closed and stapled. After surgery, rats recovered for at least 2 weeks prior to the endotoxemia experiments.

### 2.2 Experimental protocol

**FIGURE 1:**
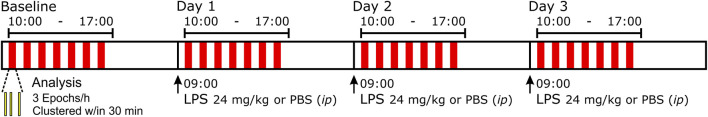
Representation of the protocol performed over four consecutive days. We placed freely moving rats in a whole-body, flow-through plethysmograph on top of telemetry plates for simultaneous breathing and blood pressure recordings over 4 sequential days. On Day 0, we recorded baseline (BSLN) activity. On Days 1–3, between 08:00–09:00 (times: 24 h clock), rats received either lipopolysaccharide (LPS) 24 mg/kg or a comparable volume of phosphate-buffered saline (PBS) intraperitoneally (*ip*). For off-line analysis, from periods of ‘stationary’ breathing, we selected three epochs (yellow-filled, black-outlined bars) that were clustered within ∼30 min (represented by red block) from each hour between 10:00 and 16:00.

Following instrumentation, we recorded the ventilatory pattern of unanesthetized freely moving rats, in a flow-through, whole-body plethysmograph (Buxco, Wilmington, North Carolina United States). To record arterial blood pressure, we placed the plethysmograph on the receiver for the implanted DSI transmitter and pressure transducer. Thus, we recorded both signals simultaneously (Spike 2, Cambridge Electronic Design, Cambridge, England). We sampled the biologic signals at 2 kHz and aimed a video camera at the plethysmographic chamber to record the rats’ behavior to help us identify non-stationaries in the record (*e.g.*, sniffing and grooming). We excluded non-stationary data from our analysis. We analyzed distinct epochs (*n* = 3, see Figure 1 and Section 2.3) from each hour that we recorded the rat during baseline and on the following three experimental days (Figure 1). During the daylong recording, we periodically removed the rats from the plethysmograph and returned them to their home cage. After recording a baseline data set, the experimental group of rats received a bolus injection of lipopolysaccharide (LPS, 24 mg/kg, *ip*, *n* = 8) and the control group received sterile phosphate-buffered saline (PBS, *ip*, *n* = 3) between 08:00 and 09:00 of Days 1–3. After LPS or PBS injections on Days 1–3, we recorded a rat in the same plethysmograph each day. For analysis, we selected 3 epochs (Figure 1 yellow-filled, black-outlined bars) in the available recordings (Figure 1 red blocks).

### 2.3 Data analysis of the cardio-respiratory waveforms

**FIGURE 2:**
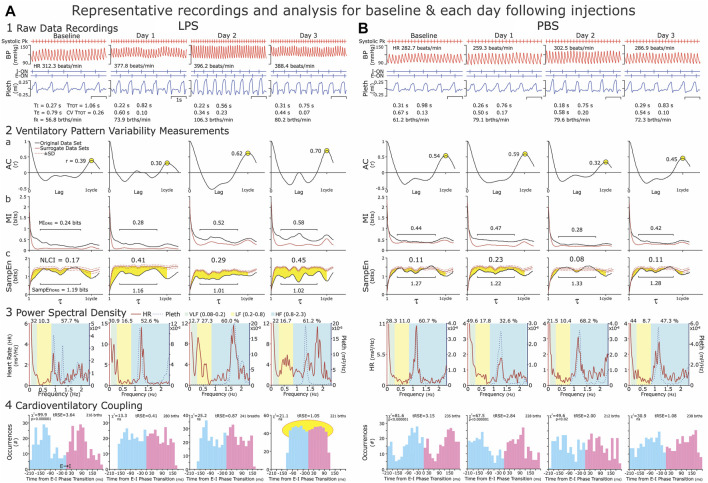
Representative recordings of arterial blood pressure and ventilatory waveforms **(Panel 1)** and illustrations of the analyses **(Panels 1–4)**. **Panel 1:** We recorded at baseline and on three consecutive days (Day 1–Day 3) following an intraperitoneal injection of LPS (Column **(A)**) or PBS (Column **(B)**). For time-domain analysis, we selected three 1-min epochs (~100 brths) from each hour of recording. Epochs occurred within 30 min but not continuous with the previous epoch. Ideally 30–40 min were between groups of epochs. We excluded sighing and the disrupted breaths immediately preceding and following sighs, as well as breathing disrupted by movements, vocalization, and sniffing, which resulted in a consistent ventilatory pattern with the CVTTOT ≤0.2. **Panel 2:** For the nonlinear analysis of ventilatory pattern variability (VPV), we calculated the Autocorrelation function (AC, Panel **(2a)**) Mutual Information (MI, Panel **(2b)**) and Sample Entropy (SampEn, Panel **(2c)**) of the original data set and 19 iAAFT surrogate data sets. We present the statistic, NLCI (nonlinear complexity index, see #s centered at the top in the graphs of Panel **(2c)**) to represent the magnitude of time-dependent variability in the respiratory airflow. The NCLI normalizes the difference between the SampEN of the original data set and mean SampEn of surrogate data sets. The greater the NLCI is, then the greater the time-dependent variability is. **Panel 3:** For the frequency analysis of heart rate and breathing waveform variability, we determined the relative Power Spectral Density (PSD) of these variables in 2-min epoch. We chose 2-min epochs, as this was the upper time limit to obtain stationary data sets consistently. **Panel 4:** Cardioventilatory Coupling (CVC) refers to the tendency of the last heartbeat in expiration to occur at a preferred latency before the onset of inspiration. We applied this analysis to the 2-min epochs because the χ2 Analysis of 200 breaths is statistically robust. These representative data reflect our understanding that systemic inflammation induced by LPS, increases in heart rate (HR) and respiratory frequency (fR) and disrupted Cardioventilatory Coupling). Both HR and fR increased modestly from Baseline to Day 1, were greatest on Days 2 or 3. We describe the data in the Results and present group data in Figure 4.

From epochs of 100 breaths, which were ∼1 min, we analyzed arterial blood pressure (BP) and plethysmographic recordings for the following time-domain variables: 1) cardiovascular variables: HR and BP, and 2) respiratory phase and cycle durations: duration of inspiration (TI), duration of expiration (TE), cycle duration (TTOT), respiratory frequency (fR) (Figure 2, Panel 1).

NLCI calculations: From the same epochs of ∼100 breaths, we measured the nonlinear dynamics of ventilatory waveform and compared Mutual Information (MI) and Sample Entropy (SampEn) of the original data to those of MI and SampEn of surrogate data sets. We constructed the surrogate data sets using the iterative amplitude-adjusted Fourier Transfer (iAAFT) method (Theiler et al., 1992; Schreiber et al., 2000). The iAAFT surrogate data sets use the amplitude distribution of the original data set and preserve the autocorrelation (AC) function (linear correlations) but disrupt the time-dependent variability of the signal (Dhingra et al., 2011). We show the autocorrelation function for original data (Figure 2, Panel 2a). MI measures the predictability of a future value given a reference value (Figure 2, Panel 2b). In contrast, SampEn measures the repeatability of 3-point patterns in a signal (Dhingra et al., 2011). In practice, a high SampEn value suggests high variability and low repeatability (Figure 2, Panel 2c). We plotted the MI and SampEn of the original (black lines) and surrogate data sets (red lines) (Figure 2, Panels 2b&c). For the ventilatory waveform, neighboring values have a high MI value which decreases as the cycle progresses (Figure 2, Panels 2b). We report the middle 65% of SampEn and MI to avoid edge effects of neighboring points and points at one cycle length (illustrated by black horizontal lines in Figure 2, Panels 2b and 2c). The NLCI is the difference between the SampEn of the original data and the mean SampEN of the 19 surrogate data sets (Figure 2 Panel 2, highlighted in yellow). We published this analytical approach in (Dhingra et al., 2011).

From epochs, that contained 200 brths (∼2 min), we calculated heart rate variability in the frequency domain. First, we identified the heartbeats from the systolic peak pressure. Second, we performed a Lomb–Scargle transformation on this time series to compensate for the unevenly sampled data (Fonseca et al., 2013). Finally, from the Lomb–Scargle periodogram, we measured power spectral density (PSD) of HR at the following frequency bands: 1) very low frequency (VLF) (0.08–0.2 Hz), 2) low-frequency (LF) (0.2–0.8 Hz) and 3) high-frequency (HF) (0.8–2.3 Hz) based on (Silva et al., 2009). The high frequency component overlaps with frequency of respiration (Figure 2, Panel 3).

From ∼2-min epochs, we measured Cardioventilatory Coupling (CVC) a second type of cardiorespiratory coupling. In CVC, we calculate the tendency for the last heartbeat in expiration to occur at a preferred latency before the onset of inspiration (Figure 2; Panel 4). For statistical robustness, the determination of CVC requires 200 breaths (Friedman et al., 2012). We plotted the distribution around the expiratory-to-inspiratory (E-I) Phase Transition and calculated the χ^*2*^ statistics. We also confirmed the significance of the distribution using a bootstrap method of comparing the χ^*2*^ distributions to surrogate data sets after 100 shuffles of the original data.

### 2.4 Data analysis of the sighs

**FIGURE 3:**
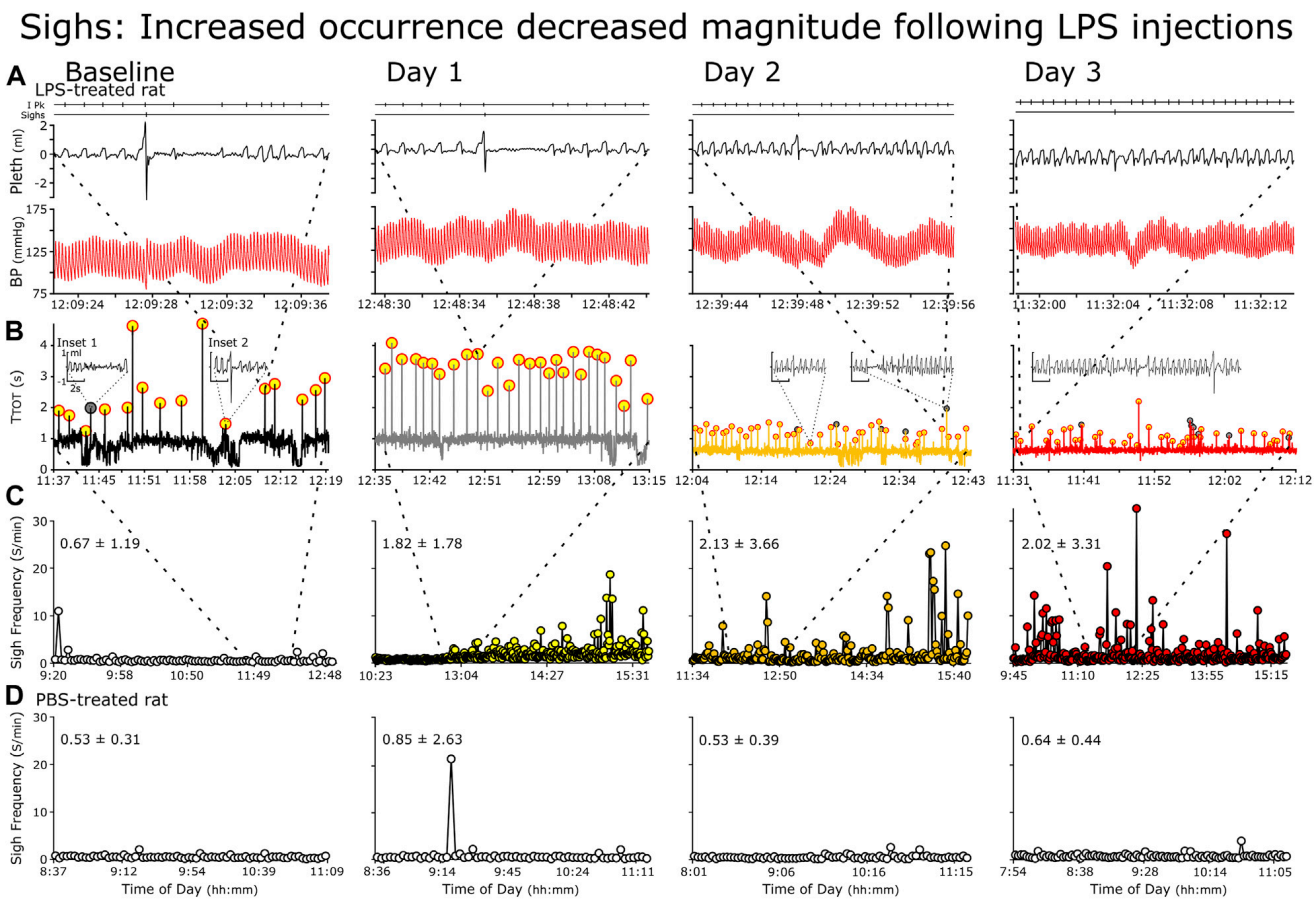
Representative changes in sighs following repetitive LPS injections. Rows **(A–C)** rats received LPS and Row **(D)**, PBS. **(A)** Individual sighs, **(B)** Successive TTOT (Cycles with sighs (highlighted in yellow with red circles) **(C**, **D)** Instantaneous sigh frequency. **(A)** Raw recording of breathing pattern and blood pressure depict a representative sigh at baseline (Left) followed by Days 1 through 3. Features of sighs at baseline include sharp increases in airflow at the end of inspiration and start of expiration and a post-sigh apnea **(B)** We plotted cycle duration against time of day. The representative sigh in **(A)** is the origin of the thick dashed lines between the panels, the dotted lines mark the location of the raw record within the panels. Generally, cycle duration increases during sighs (highlighted in yellow) due mainly to a prolonged expiration. However, an increase in cycle length occurs with spontaneous apneas (gray circles and Inset 1). Finally, sighs can occur with state transitions, especially from sleep to arousal (Inset 2 arousal evident from the increased fR and an increased variability in fR). **C** We plotted instantaneous sigh frequency (fS) against time in the record to demonstrate the increase in the incidence of sighs. The breaths for the cycle-by-cycle plot of TTOT is mark by the dashed lines between panels.

**FIGURE 4:**
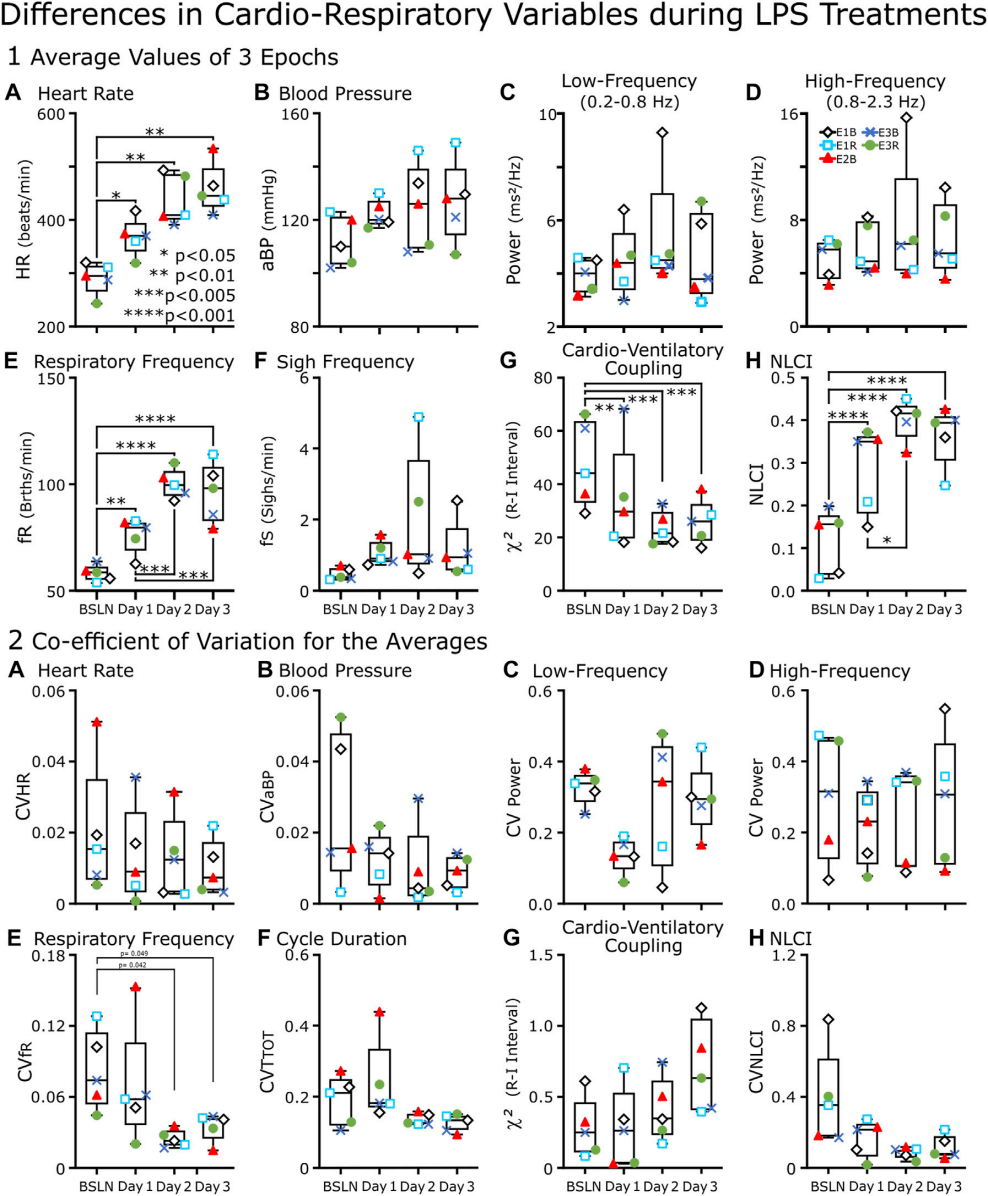
Box-and-whisker plots of cardiorespiratory variables of LPS-treated rats (*n* = 5) from baseline through Day 3.**(A–H)** Each point represents an average of the values from three epochs of data recorded between 11:00–13:00. An epoch was either 1 min (∼100 breaths) **(A**, **B**, and **(E))** or ∼2 min (200 breaths) (**C**, **D**, **G**, and **(H)**. In **(F)**, the point represents the average number of sighs per minute for the recording period between 11:00 and 13:00. **(A)** Heart Rate (HR), **(E)** Respiratory Frequency (fR) and **(H)** Nonlinear Complexity Index (NLCI) increased from Baseline (BSLN); whereas **(G)** Cardioventilatory Coupling (χ^*2*^ values) decreased. In contrast, the following variables did not change significantly for the group: **(B)** Blood Pressure (BP), the **(C)** low- and **(D)** high-frequency components of the power spectral analysis and **(F)** Sigh Frequency (fS). **(A–H)** Each point represents the coefficient of variation (CV) of the mean from three epochs of data recorded between 11:00–13:00. In **(F)**, we replaced fS with Cycle Duration (TTOT) for because 1) fS was not a mean of three epochs rather the mean of the recorded period and 2) CVfR had a weak but significant (*p* < 0.05) decrease in Days 3 and 4 compared to baseline. This was not evident in CVTTOT.

In the analysis of the ventilatory pattern in the LPS-treated rats, generally, sigh frequency (fS) increased and the sigh waveform morphed (Figure 3). Specifically, the magnitude of the augmented breath decreased, e.g., the secondary inspiratory flow, the expiratory flow and the post-sigh apnea decreased (Figure 3). We present fS (Figure 4 Panel F).

### 2.5 Statistical differences and Correlations between variables

We used Pearson test to analyze data for correlations (GraphPad Prism version 9.3.1 for Windows, GraphPad Software, San Diego, CA, United States). To determine statistical significance, we performed two-way repeated measures ANOVA followed by Student-Newman-Keuls test with statistical significance of *p*-value <0.05 (SigmaStat, v 4.0, Systat Software, San Jose, CA). To allow for acclimation to the plethysmograph and to the PBS or LPS injections, we performed the initial statistical tests on data from the mid-day recording sessions (11:00–13:00). We were able to recover only 5 of the 8 Baseline recordings in the LPS group. Consequently, the statistical comparison within the LPS group were based on these 5 rats. A two-way ANOVA was performed on rats comparing BSLN to Days 1–3.

### 2.6 Lung Pathology and Cytokine measurements

Briefly, we over anesthetized the rats, inflated their lungs and perfused them with 10% formalin for 30 min (Jacono et al., 2011). We removed the lungs, transferred them to a cassette and embedded them in paraffin. We cut 5-μm sections and stained the lungs with hematoxylin and eosin to determine if lung injury was evident after the LPS injection protocol.

In a subset of rats (*n* = 2), we inflated the lungs, perfused the rats and processed the lung to assess tissue injury. In others (*n* = 8) we removed unfixed lung tissue, homogenized it in complete lysis buffer (Roche Complete Lysis-M Buffer, Cell Signaling Technology, Danvers, MA, United States) and centrifuged. We measured cytokine concentrations (IL-1β, IL-6 and TNF-α) in supernatants of homogenized lungs by quantitative sandwich enzyme immunoassay technique (ELISA) (R&D Systems). We pipetted standards and samples to microplate wells pre-coated with a monoclonal antibody specific for the relevant cytokine. After washing, we added an enzyme-linked polyclonal antibody specific to the cytokine. After washing again, we added substrate solution and the amount of bound cytokine quantified spectrophotometrically.

### 2.7 Immunohistochemistry of brainstem

After sacrificing and perfusing the rats with 4% paraformaldehyde (*n* = 2), we harvested the brainstem (Jacono et al., 2011). We cryoprotected the brainstem in 15% sucrose for ∼12 h and then in 30% sucrose until the tissue sank (24 h). We blocked the brainstems, embedded the tissue in freezing medium (Triangle Biomedical Sciences) and cut frozen sections (20 μm) using a cryostat (Leica). We collected every fourth section on a different slide. The slides stored at −20°C. We stained a set of slides for IL-1β (Abcam) with a set of slides serving as a negative control (no primary antibody). The procedure followed these steps: 1) 0.03% H_2_O_2_ in sodium phosphate buffer (PBS), 30 min; 2) 20% bovine serum albumin, 3% Triton X-100 in PBS, 60 min; 3) 1:100 dilution of primary antibody, 0.01% Triton X-100, 5% goat serum in PBS, overnight; 4) Three washes in PBS for 10 min; 5) 1:200 dilution of biotin conjugated secondary antibody (goat anti-rabbit, Jackson Labs), 0.01% Triton X-100, 5% goat serum, 2 h; 6) Three washes in PBS for 10 min; 7) avidin–biotin complex (ABC kit standard elite, Vector) 30 min; 8) Three washes in PBS for 10 min; 9) 0.03% 3,3′-diaminobenzidine, 0.03% NiCl2, 0.008% H_2_O_2_, 6–10 min; 10) Three washes in PBS for 5 min). Finally, we dehydrated the sections by immersing them in solutions with progressively greater alcohols and xylene. We applied a drop of permount, and cover slipped the slides.

## 3 Results

### 3.1 Cardiovascular and respiratory responses to LPS

**FIGURE 5:**
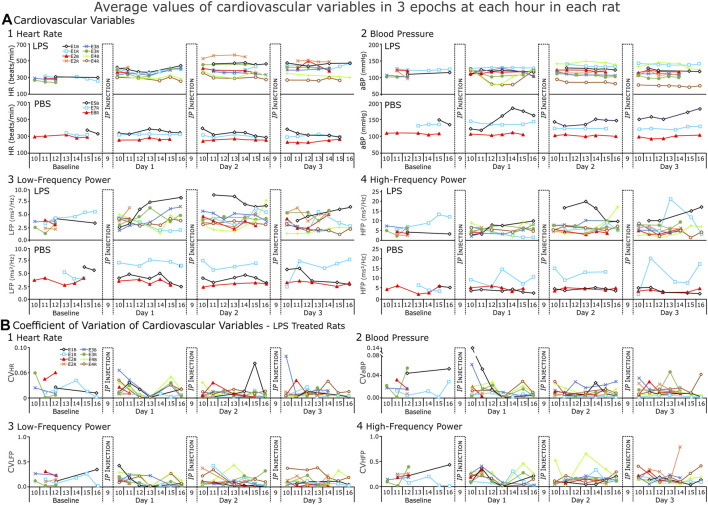
Cardiovascular variables from each rat (LPS = 8, PBS = 3) for each day of the recording. **(A)** Average values and **(B)** Coefficient of variation (CV) for those values from LPS-treated rats. In **(A)**, each point of heart rate (HR) blood pressure (BP), low- and high-frequency power (LFP and HFP respectively) represents the average of three epochs. The CV indicates low variation (predominantly <0.2) in the repeated measures from each hour. The increase in the range of values during the response to LPS reflects inter-rat variation.

**FIGURE 6:**
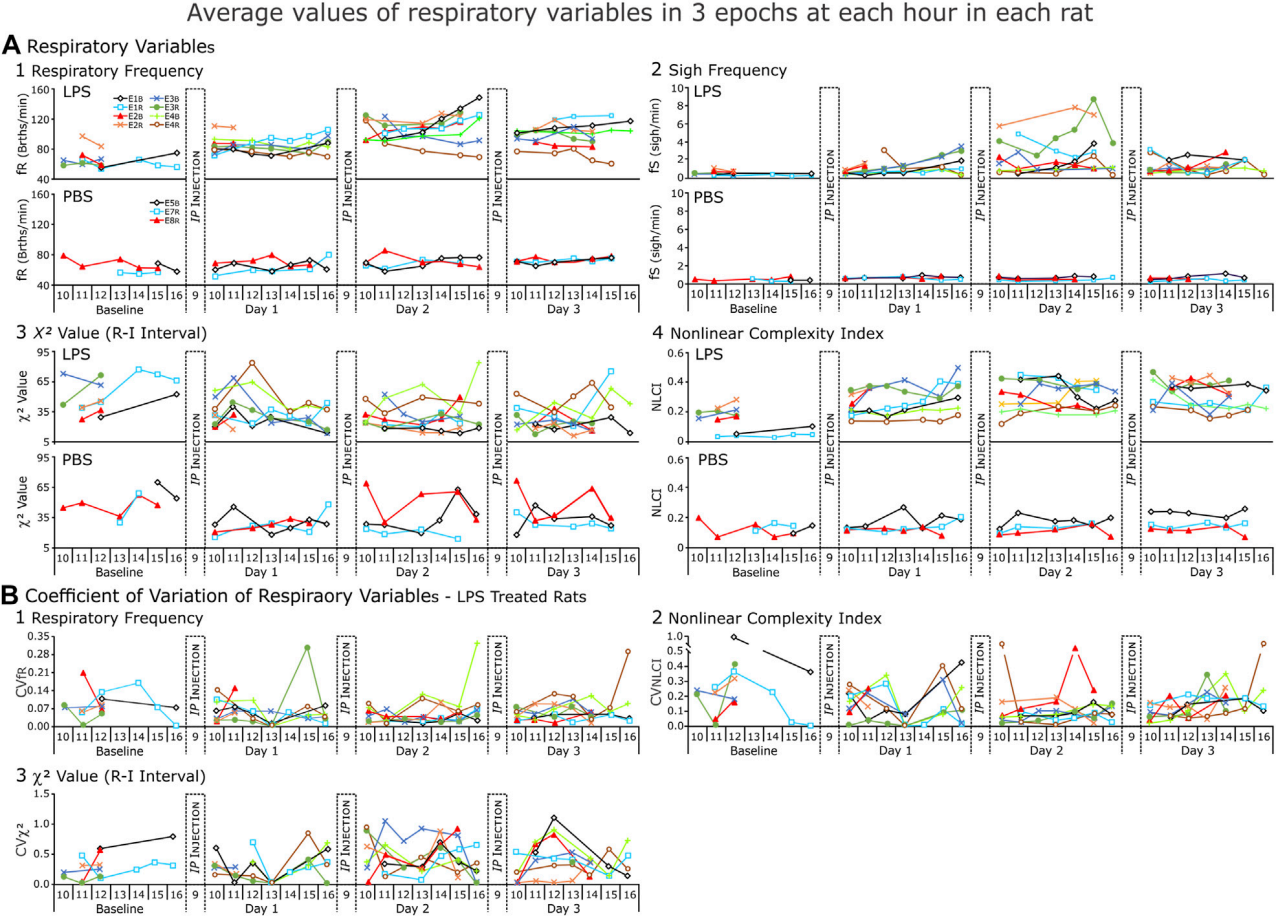
Respiratory variables from each rat (LPS = 8, PBS = 3) for each day of the recording. **(A)**. Average values and **(B)** Coefficient of variation (CV) for those values. In **(A)**, each point of respiratory frequency and nonlinear complexity index represents the average of 3 1-min epochs; of χ^*2*^ value, an average of 3,200-breath epochs for each hour; and sigh frequency (fS), average instantaneous fS (sighs per minute) present in each hour of recording. In **(B)**., each point represents coefficient of variation of the averages. NOTE: the *y*-axis changes with each variable but is consistent from Baseline to Day 3.

**FIGURE 7:**
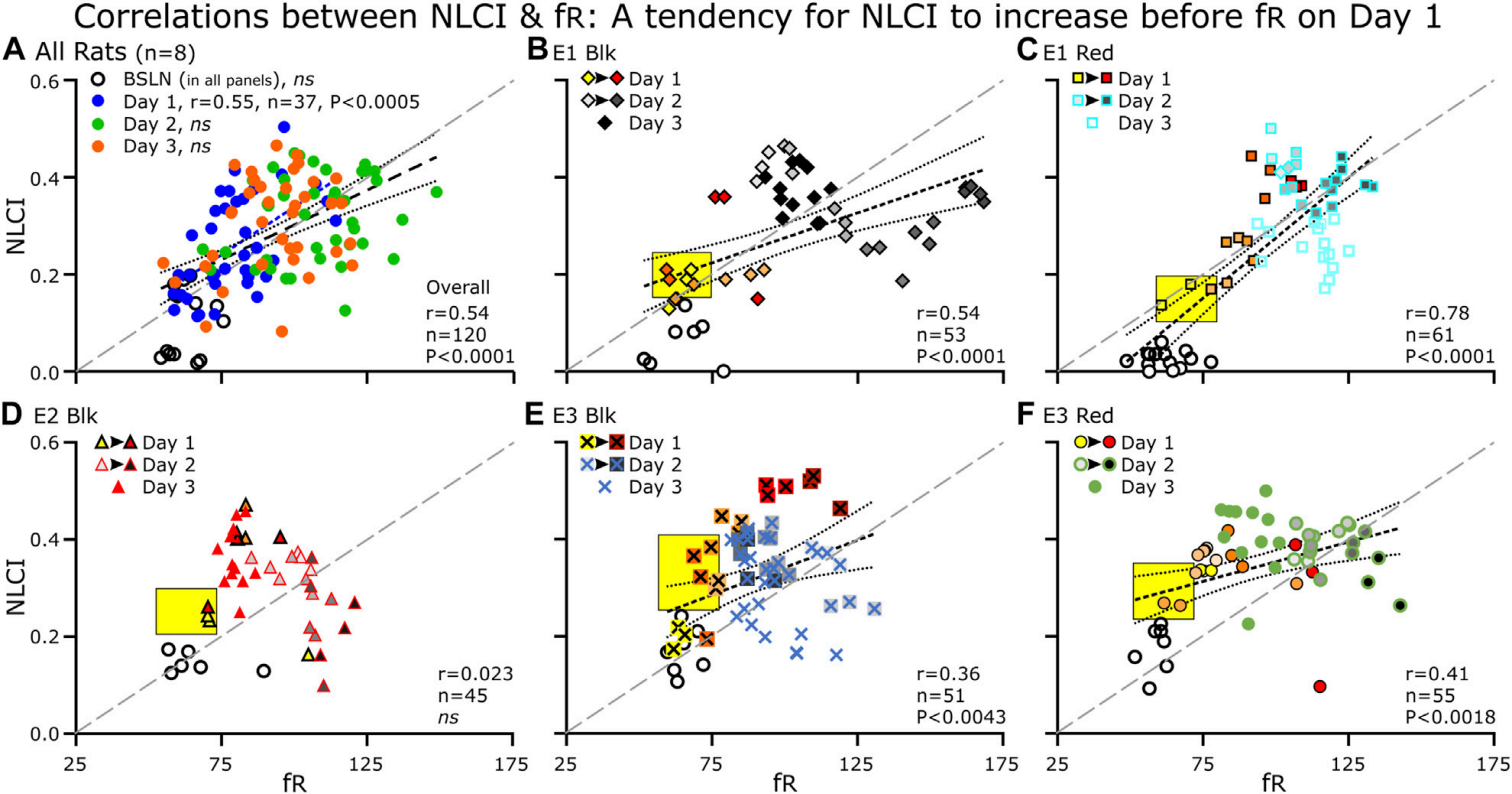
Correlations between NLCI and fR for the group and for individual rats at each day (BSLN and Days 1–3). **(A)** For the group data (*n* = 8), each point represents the average of 3–5 60-s epochs. **(B**–**F)** for the individual rats (*n* = 5), each point represents a single epoch. For Days 1 and 2, we color-coded epochs from the same hour of the day with the earliest hour being the lightest color (Day 1: fill, Yellow at 10:00 to Dark Red at 16:00) or shade of gray (Day 2: fill, Light Gray to Black). For Day 3, we used only one symbol. **(B–F)** The yellow black-bordered box highlights increases in NLCI occurring before increases in fR. **(A–F)** A diagonal line (long-dashed, gray line) separates the points below in which the relative increase in fR is greater than that of NLCI from the points above in which the relative increase in NLCI is greater than the that of fR. **(A–F)** at BSLN (open symbols), NLCI was below 0.2 and was close to zero in two rats **(B, C)**. **(B–F)** On Day 1, NLCI increased prior to an increase in fR (Yellow boxes). NLCI increased initially during the day (light yellow to red symbols) but decreased by late afternoon (dark red symbols) **A** NLCI was correlated to fR across days (blue dashed line with long dashes) and on Day 1. **(B**, **C**, **E** and **F)** The correlation between NLCI and fR was apparent (a dark dashed line). Generally, Day 2 has the highest fR and high NLCI and on Day 3, points clustered in the middle of the correlation.

**FIGURE 8:**
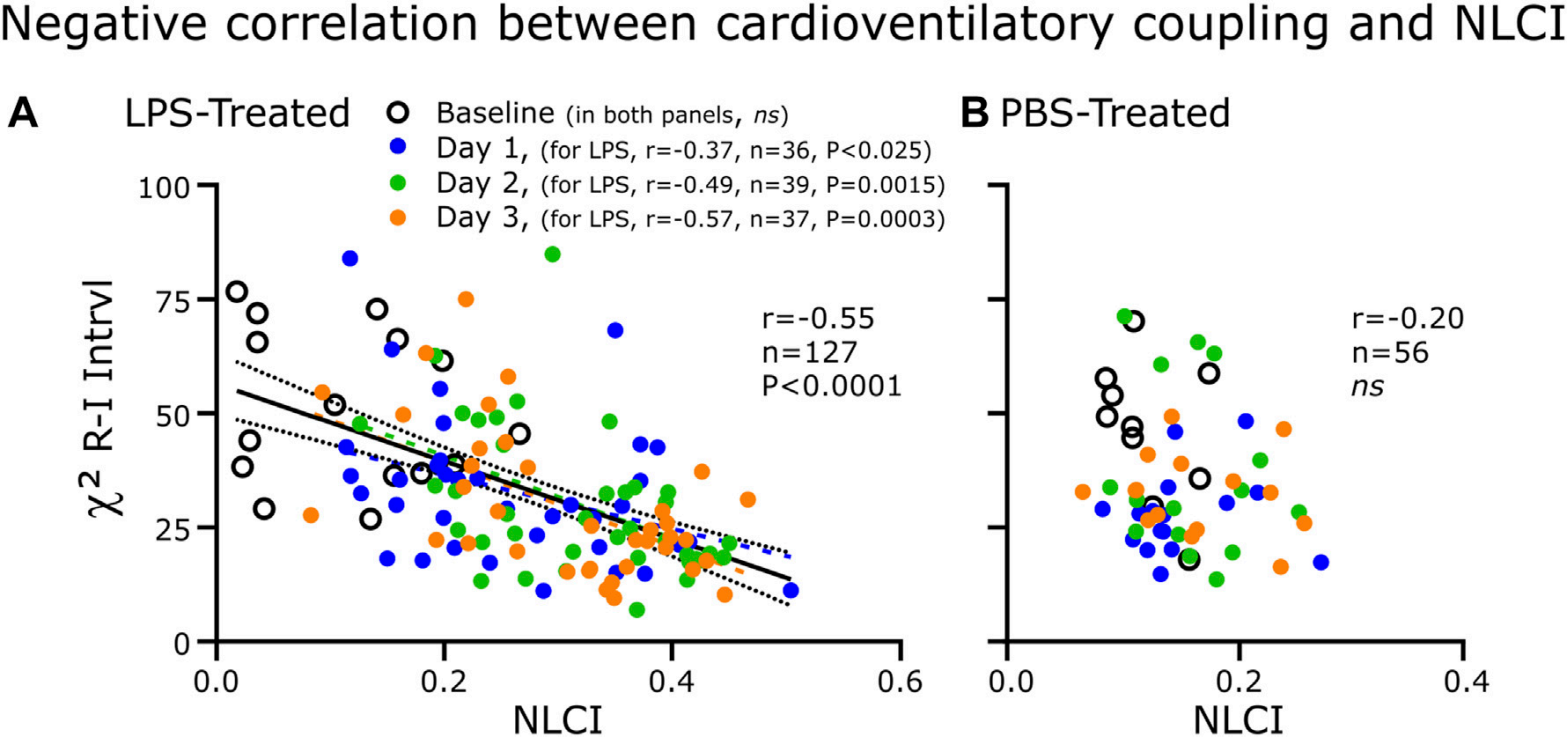
Correlations between CVC (χ^2^) and NLCI for the LPS-treated **(A)** and PBS-treated **(B)** groups. Each point represents the 200-breath epochs. **(A)** For the LPS-treated group (*n* = 8), CVC decreased as NLCI increased. The strength of the negative correlation increased progressively from absent at Baseline to becoming greater following each injection of LPS. **(B)** In the PBS-treated rats (*n* = 3), CVC was not correlated to NLCI.

To quantify the cardiovascular and respiratory response to LPS injections, we plotted the averages from three epochs recorded at noon hour on each day from five rats (box-and-whisker plots Figure 4A) and in an accompanying plot (Figure 4B), we graphed the coefficient of variation for each of the averages. Figure 4 serves is a cross-section for the next two plots (Figures 5, 6). In Figure 5, we plotted only the cardiovascular variables recorded in each rat at various hours on each day (Figures 5, 6), we plotted these points for the primarily respiratory variables. We did this for the following reasons: 1) a few rats had distinctly different dynamics in the response to the LPS injections and 2) recordings were not available for every rat at each time point; for example, we could not retrieve baseline data from 3 rats. Finally, the variance in the responses did provide the opportunity to correlate variables (Figures 7, 8).

Box-and-whisker plots of cardiorespiratory variables show baseline (BSLN) and the response approximately 4 h after LPS injections on Days1–3 (Figure 4). The primary comparisons using a one-way repeated measures ANOVA were between BSLN and the days following LPS Injection (*n* = 5). In this comparison respiratory frequency (fR), heart rate (HR), and NLCI increased, while χ^*2*^ values decreased from baseline on each day following injections. Secondary comparisons are between injection days. In this comparison, only respiratory variables changed significantly; both fR and NLCI increased on Day 2 compared to Day 1. Only fR remained increased from Day 1 on Day 3.

### 3.2 Expanded comparisons of cardiovascular responses to LPS and PBS treatments

We performed two-way repeated measures ANOVA to compare the response to LPS injections to that of PBS injections. Note, for the LPS Group, *n* = 5 at BSLN and *n* = 8 on Days 1–3, while for the PBS Group *n* = 3 (Figures 5, 6).

We expected HR to increase in LPS-treated rats (Consistent with Figure 4) and not to change in PBS-treated rats (Figure 5A1). However, even though HR increased in the first hours after LPS injections, HR decreased progressively in the afternoon in two rats. By the end of Day 1, HR was not significant increased over BSLN because the range of HRs increased. As compared to HR at BSLN (291.0 ± 29.9 bpm), the majority of rats increased their HR in response to LPS on Day 1 (377.0 ± 23.1 bpm), on Day 2 (433.0 ± 51.8 bpm) and on Day 3 (458.0 ± 45.1 bpm). In the PBS-treated group, HR did not differ from BSLN (321.0 ± 35.0 bpm) compared to Day 1 (308.0 ± 42.0 bpm), Day 2 (322.0 ± 28.7 bpm) or Day 3 (333.0 ± 41.0 bpm).

We anticipated that BP should not change in PBS-treated rats. In contrast, we expected that changes in BP could be biphasic in this LPS model due to 1) an initial increase due to an activation of sympathetic nerve activity, and 2) a decrease in rats with severe septicemia due to increased vascular permeability and vasodilation (Figure 5A2). However, BP responses varied across rats after the LPS-injections. Compared to BSLN (112.00 ± 9.44 mmHg, *n* = 5), BP increased in most rats (*n* = 6 of 8) on Day 1 (122.00 ± 5.32 mmHg), Day 2 (125.00 ± 15.90 mmHg) and Day 3 (127.00 ± 15.10 mmHg). Like HR, the increase in the range of BP values was a salient feature. In two rats on Day 1, BP decreased from 10:00 but recovered by 16:00 (10:00, 125.00 ± 0.30 mmHg; 12:00, 79.40 ± 1.39 mmHg; 14:00, 79.50 ± 1.13 mmHg; 15:00, 102.00 ± 32.00 mmHg; and 16:00, 121.00 ± 0.10 mmHg). This occurred consistently as on Day 2, BP decreased after the injection; and on Day 3, BP remained low in one and decreased in the other. Differences in BP (independent of the PBS injections) were apparent in the PBS-treated group (*n* = 3). One rat had a consistently low BP (∼110 mmHg). The other two rats had a consistently high BP (∼150, ∼140 mmHg).

Average HRV in the low- and high-frequency bands (LFB and HFB respectively) had narrow ranges at BSLN prior to either LPS- and PBS- treatment (LFB, 2.30–5.65 Hz; HFB, 2.30–8.91 Hz) (Figure 5A3, A4; Figure 5B). On Day 1 in the LPS-treated rats, the LFB varied and had a range compared to the BSLN (LFB, 2.81–5.82 Hz; Figure 5A3) whereas the HFB consolidated to a narrow range (HFB, 3.46–6.86 Hz; Figure 5A4). On Days2and3 in the LPS-treated rats, the range of the LFB and HFB increased (Day 2, LFB, 3.52–7.93 Hz; HFB, 3.64–13.40 Hz; Day 3, LFB, 2.99–6.48 Hz; HFB, 4.54–13.40 Hz). In the PBS-treated rats on Days 1–3, the LFB remained close to BSLN but the HFB increased in one rat from 4.63 Hz at BSLN to 9.69 Hz (Day 1) to 12.5 Hz (Day 2) to 10.7 Hz (Day 3).

An ideal biometric would provide consistent measures of variables in similar state space and be responsive to state changes. In Figure 5B, we plot the coefficient of variation (CV) for each of the averages in Figure 5A. The CVs were low for repeated measures of HR (Figure 5B1) and BP (Figure 5B2). In contrast, CVs for repeated measures of the actual power of the LF and HF bands were often (∼30%) >0.25.

### 3.3 Expanded comparisons of respiratory responses to LPS and PBS treatment

We expected respiratory (fR) and sigh (fS) frequencies to increase in LPS-treated rats and not to change in PBS-treated rats (Figure 6A). In general, fR increased from BSLN as early as noon on Day 1 and remained elevated on Days 2 and 3 (*n* = 5 rats: BSLN, 58.3 ± 3.8 breaths per minute (BrthsPM); Day 1, 80.1 ± 12.2 BrthsPM; 111.2 ± 7.0 BrthsPM on Day 2, and 96.0 ± 13.9 BrthsPM on Day 3) in the LPS group. In contrast, fR remained unchanged in the PBS-treated group: at Baseline, 64.7 ± 8.6 BrthsPM; Day 1, 66.8 ± 5.5 BrthsPM; Day 2, 66.7 ± 14.9 BrthsPM; and Day 3, 68.8 ± 0.1 BrthsPM; Figure 6A.

Unlike the fR, fS increased for only a subset of the LPS-treated group (in 4 of 8 rats; Figure 6B). If fS increased, the change occurred later than the increase in fR in the afternoon on Day 1, highest on Day 2 and decreasing by Day 3. For example, in Figure 3, we show a rat following LPS-treatment, the magnitudes of the inspiratory and expiratory flows in the representative sigh decreased on Day 1 and decreased more on Days 2 and 3 whilst the duration of the post-sigh apnea decreased on Days 2 and 3. In contrast, even though the sighs appeared diminished, it had a greater transient effect on blood pressure, which was most apparent on Day 3. The averages for *n* = 4 are: BSLN, 0.525 ± 0.119 Sighs per min (SighsPM); Day 1 at 15:00–16:00, 2.350 ± 0.612 SighsPM; Day 2 at 13:00–16:00, 4.360 ± 2.430 SighsPM; and Day 3, 1.220 ± 0.620 SighsPM. In the PBS-treated group, fS remained low as follows: BSLN, 0.516 ± 0.128 SighsPM; Day 1, 0.629 ± 0.035 SighsPM; Day 2, 0.477 ± 0.157 SighsPM; and Day 3, 6.010 ± 0.241 SighsPM.

We expected CVC as measured by χ^2^ values to decrease in the LPS-treated group (Figure 6A). However, CVC was not uniformly high at Baseline (47.5 ± 16.0). Nevertheless, at noon on Day 1, CVC values decreased in 4 of 5 rats (34.4 ± 20.1). By Day 2, CVC had decreased at midday (23.4 ± 6.4) and remained low on Day 3 (25.7 ± 7.9). Higher values occurred later in the day.

The NLCI is an index of the predictability of pattern of 3 points and is normally ≤0.2 in a healthy rat (Jacono et al., 2011; Chung et al., 2013; Horton et al., 2022) and we expected NLCI to increase to reflect an increase in the predictability of the pattern following LPS. An increase in NLCI compared to Baseline was evident by noon on Day 1 and then NCLI increased further by noon on Day 2 and remained increased on Day 3 (Baseline, 0.117 ± 0.076; Day 1, 0.288 ± 0.101; Day 2, 0.402 ± 0.048; Day 3, 0.365 ± 0.070). For the PBS-treated group, NLCI remained ≤0.2 (Baseline, 0.109 ± 0.020; Day 1, 0.131 ± 0.019; Day 2, 0.177 ± 0.073; Day 3, 0.156 ± 0.065).

We plotted CVs for the means of fR, NLCI, and χ^2^ values (Figure 6B). We expected CV to decrease for fR and NLCI following repeated LPS injections, because as these values would increase a ceiling effect would limit their variability. In most cases, this happened, but one of the two exceptions occurred for NLCI in the sickest rat as it was dying.

### 3.4 Correlation between NLCI and fR after LPS or PBS treatment

An increase in NLCI compared to Baseline was evident at noon on Day 1 and then NCLI increased further at noon on Day 2 and remained increased on Day 3 (Baseline, 0.117 ± 0.076; Day 1, 0.288 ± 0.101; Day 2, 0.402 ± 0.048; Day 3, 0.365 ± 0.070). For the PBS-treated group, NLCI remained ≤0.2 (Baseline, 0.109 ± 0.020; Day 1, 0.131 ± 0.019; Day 2, 0.177 ± 0.073; Day 3, 0.156 ± 0.065).

For the LPS-treated Group on Day 1, NLCI was correlated positively to fR (*r* = 0.54, *p* = <0.0001), which was evident on Day 1 (*r* = 0.55, *p* = <0.0005) but not at Baseline (*r* = 0.15, *p* = 0.62), Day 2 (*r* = 0.22, *p* = 0.17) nor Day 3 (*r* = 0.25, 0.12) (Figure 7A). For the five individual rats with Baseline recordings; when combining all the days, NLCI was correlated to fR a four rats (Figures 7B, C, E, F) but not for the 5th one (Figure 7D). In the PBS-treated group, NLCI did not correlate with fR (*r* = 0.034, *ns*).

As we expected, Figures 7A–F NLCI at baseline (open symbols), was below 0.2 and were close to zero in two rats (B&C). On Day 1, we expected an increase in NLCI: that would occur over time, that NLCI would increase prior to an increase in fR, and that NLCI would decrease by the end of the recording on Day 1. In A, the correlation (blue dashed line) between NLCI and fR on Day 1 was above the diagonal line. In B, C, E and F an arrow indicates the trajectory of the points. Generally, the last point of the day has the highest NLCI (and fR) the exception is B, E1-Blk. Day 2 has the highest fR and high NLCI. On Day 3, points clustered in the middle of the correlation. D. In rat E2-Blk even though NLCI did not correlate to fR, general properties described for the other rats were evident.

### 3.5 Correlation between CVC and NLCI after LPS or PBS treatment

We expected CVC as measured by χ^2^ to be correlated negatively to NLCI in the LPS-treated group (Figure 8). We theorized that with LPS-induced brainstem inflammation would decrease the effectiveness of baroreceptor input on altering the pattern generator. Not only did CVC correlate negatively to NLCI but also this correlation strengthened progressively with each day of LPS treatment. In the PBS-treated group, CVC did not correlate to NLCI.

### 3.6 Peripheral inflammation: Lung and serum

**FIGURE 9:**
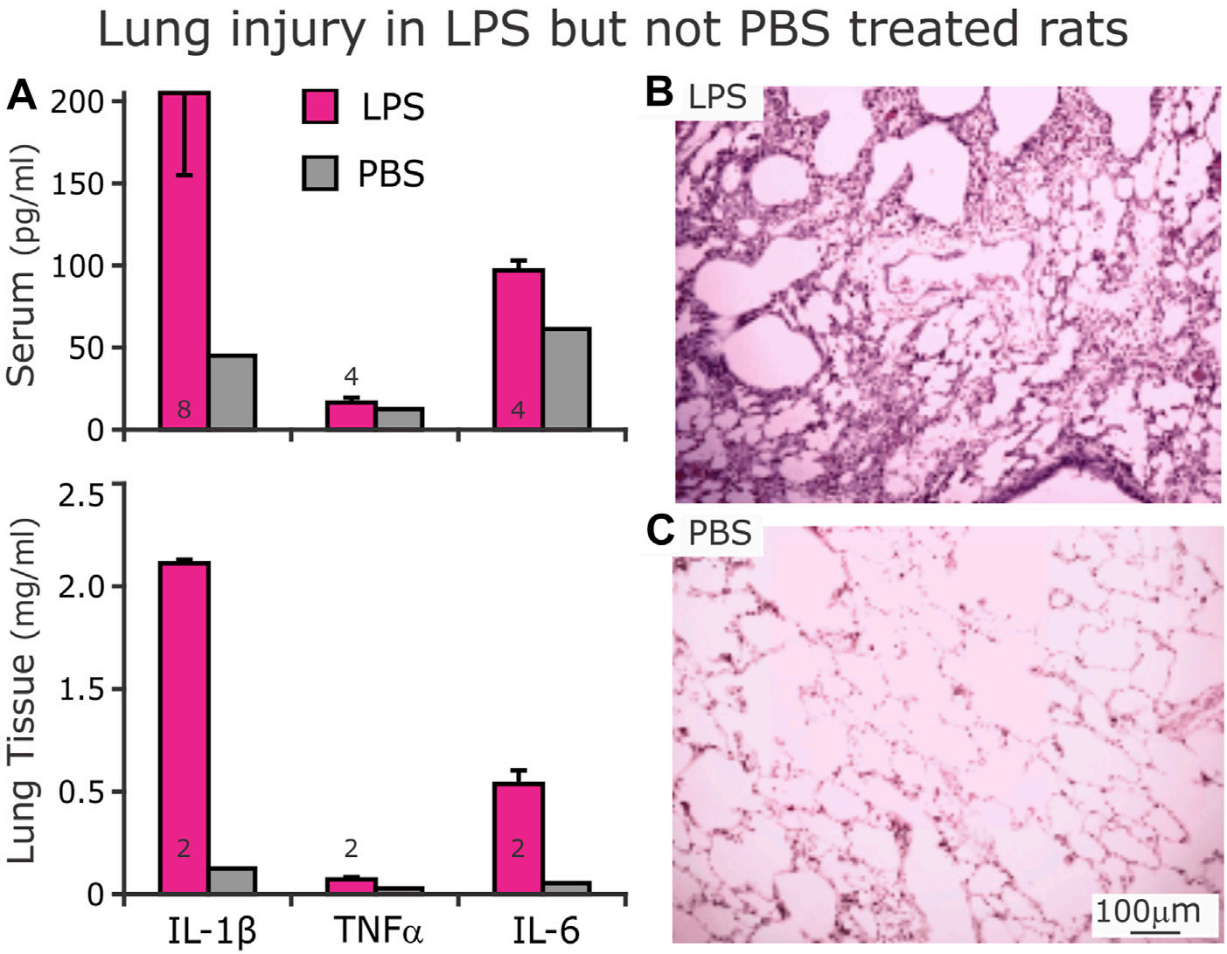
Expression of proinflammatory cytokines in serum (**(A)**, Top) and lung homogenates (**(A)**, Bottom) after D3 of either LPS (red bars) or PBS (gray bars, n = 1) injections. **(A)** After 3 days of LPS injections, serum (above) concentrations of IL-1β and TNFα were not different from their respective serum concentrations from rats that received PBS injections. However, serum IL-6 was higher from LPS- compared to PBS- treated rats. In homogenized lung tissue, IL-1β and IL-6 were higher in LPS- than PBS- treated rats. In contrast, the lung tissue had low levels TNFα. **(B)** Representative histologic section of lung tissue from LPS-treated rat showing cellular infiltrates with hyaline membranes bordering airways. **(C)** Histologic section of lung tissue from a PBS-treated rat showing clear alveoli.

The serum had increased proinflammatory cytokines IL-1β, TNF-α and IL-6 after 3 days of LPS injections *ip* compared to the PBS-treated group (Figure 9A, Top). Further, representative histological tissue sections from the lungs of LPS-treated (Figure 9B) compared to the PBS-treated (Figure 9C) rats reveal tissue damage in the LPS-treated lungs. Finally, increases in proinflammatory cytokines in the homogenized lungs in LPS-treated rats confirm the injury as well (Figure 9A, Bottom).

### 3.7 Central inflammation: Dorsolateral (dl) Pons and dorsolateral (dl) medulla

**FIGURE 10:**
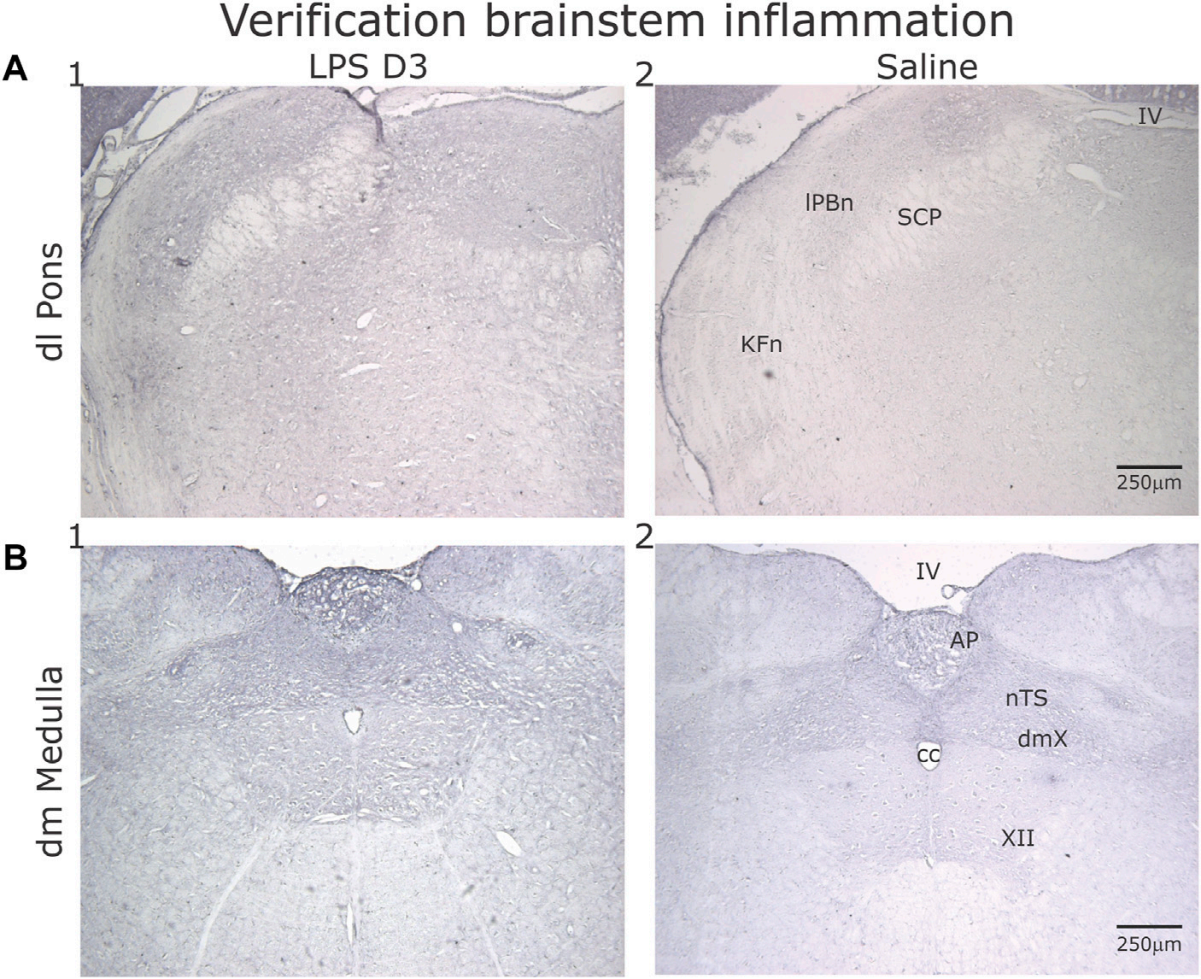
Representative sections showing IL-1β, staining in the dorsolateral (dl) Pons **(A)** and dorsomedial (dm) Medulla **(B)** after D3 of either LPS (1) or PBS (2) injections. **(A)** Comparing the LPS- to the PBS- injected rats, increased IL-1β expression was evident in the Kӧlliker-Fuse nucleus (KFn) and lateral parabrachial nucleus (lPBn). **(B)** Similarly, both the area postrema (AP) and the nucleus tractus solitarii (nTS) displayed increased IL-1β expression.

Increased IL-1β staining was present in the dorso-lateral (dl) pons and dorsomedial (dm) medulla in the LPS- (*n* = 2, Figure 10A1, 10B1) treated compared to the PBS- treated group (*n* = 2, Figure 10A2, 10B2).

## 4 Discussion

This study details the effect of systemic inflammation on the centrally generated patterns of the cardiorespiratory systems that maintain homeostasis. While the dynamics of the results vary among the rats, the correlations between variables reveal consistent interactions possibly from a common source. Further, the consistency and repeatability NLCI measurements support our hypothesis that NLCI complements HR, BP, and fR as biometrics in determining the onset of septicemia.

On Day 1 after the LPS injection, the initial effect was as an increase in the predictability of the ventilatory pattern that occurred as early as 2 h post-injection. The accompanying increase fR occurred later. On Day 2 after the second LPS injection, the highest values of NLCI, fR, fS, HR, and BP occurred; but at different times. HR and BP were highest at midday, whereas fR and fS were highest in the late afternoon. The time and day of the highest value for NLCI varied across rats. Finally, CVC correlated negatively to NLCI, and generally, χ^2^ values were the lowest on Day 3.

### 4.1 Cardiovascular response to systemic inflammation

In the hospital setting, sepsis screening tools have focused on increases in HR, fR and white blood cell (WBC) count. During systemic inflammation, the autonomic nervous system alters HR, vasculature tone and HRV (Carrara et al., 2020; 2021). High HR (>90 bpm in adult humans) is a recognized as an early warning sign of sepsis (Gyang et al., 2015). We observed increased HR after LPS injections as early as the afternoon on Day 1 and during Days 2 and 3.

In contrast to HR, HRV especially measured by power spectral analysis, has recently developed a controversial history. In the last 40 years, the relative power of the HF band (rHFP) was recognized as an index of respiratory-modulated vagal efferent activity. The LF band was theorized to be a complex interaction of vagal and sympathetic tone associated with the baroreflex control of HR. Clinically, decreased HRV associated with systemic inflammation and was related to decreased rHFP due (Buchman et al., 2020) and baroreceptor dysfunction (Annane et al., 1999). The LF/HF ratio of HRV was interpreted as a measure of sympathetic nerve activity. This is a controversial aspect of the frequency domain measures of HRV. Many colleagues do not support its use as an index of cardiac sympathetic nerve activity. For instance, Dr. Billman (Billman, 2013) wrote “The LF/HF ratio does not accurately measure cardiac sympatho-vagal balance.” Further, a convened NIH committee wrote that what is needed is to “Improve the specificity of noninvasive measures of SNA (i.e., HRV measures do not adequately represent the sympathetic component)” (Mehra et al., 2022).

In our study, both LFP and HFP varied after LPS injections. We found no statistical difference in either in the paired comparison across days post-injection. However, multiscale entropy, a nonlinear measure of HR, has promise as a biometric (Vandendriessche et al., 2014; 2017b).

Systemic inflammation occurs during early stages of sepsis. Other biomarkers used clinically to indicate infection are fever, high WBC count (>12 K), and increases in C-reactive protein. Fever is an energetically expensive process, induced by the central nervous system and is a component of ‘sickness behavior’. Additional peripheral components mediated by the sympathetic nervous system and affected by sepsis include vasoconstriction and sweating (Blomqvist and Engblom, 2018; Carrara et al., 2020). Also, excess serum C-reactive protein increases blood pressure directly (Smith et al., 2005).

In our LPS-treated group, most (6 of 8) rats had higher a blood pressure on Days 1–3 than that at baseline and that of PBS-treated group, which is consistent with increased sympathetic nerve activity and serum C-reactive protein. Nevertheless, the observed increase in BP was not consistent, generally not as robust as the increase in HR, and thus, not considered as a biomarker.

### 4.2 Respiratory response to systemic inflammation

Respiration in healthy individuals is stable, yet flexible and responsive, acting to maintain homeostasis. A high fR (>20 brthspm in adult humans) is included on sepsis screening tools. In the LPS group, fR increased from baseline on Day 1, peaked on Day 2 and remained elevated through Day 3. An increase in metabolism in the acute phase of sepsis may underlie the increase in fR (Kamisoglu et al., 2015; Wasyluk and Zwolak, 2021). Further inflammation affects lung dynamics resulting abnormal gas exchange contributing to the metabolic load (Zila and Calkovska, 2011; Hotchkiss et al., 2016).

Further, a motivation for evaluating respiration as a biomarker was a 2014 report of Seely et al., which asked, “Do heart and respiratory rate variability (RRV) improve prediction of extubation outcomes in critically ill patients?” Even though altered HRV and RRV (during spontaneous breathing trials (SBT) prior to extubation) were associated with extubation failure, a predictive model using RRV during the SBT projected respiratory failure with the greater accuracy than HRV. We hypothesized that our nonlinear-based analysis would outperform HRV and serve as an effective biometric.

In contrast to fR, fS is not used as a biomarker clinically, we observed dramatic increases in sighing but it was not present in all the rats and, thus, the fS increase was not significant. Brainstem inflammation and the associated production of prostaglandin E2 (a prostanoid involved in the induction of inflammation) may have a role in the increase of fS. In an *in vitro* study, injections of PGE_2_ in the preBötzinger Complex at low concentrations increased fS (Koch et al., 2015). Further, the CNS does produce PGE_2_ to mediate fever in response to inflammation. In our study, while fS increased greatly in a subset of rats, with Day 2 rats having rates as high as 8 sighs per minute, it waned by Day 3.

Mammalian ventilation exhibits complexity in the structure of its variability (Costa et al., 2005; Wysocki et al., 2006; Dhingra et al., 2011). Ventilation depends on extrinsic factors like room temperature, ambient oxygen, and altitude; and on intrinsic factors like body habitus, blood flow, and neural sensing (Bruce, 1996). These factors affect variability two ways: 1) randomly or stochastically and 2) nonlinearly or deterministically. Using surrogate data sets that shuffle the original data but retain the autocorrelogram disrupt the time-dependent variability but retain the linear structure of the original data set. Thus, in SampEn, the difference between the original and surrogate data sets in the predictability of a 3-point pattern reflects the nonlinear deterministic component of the waveform, which in our case, is ventilatory waveform measured by plethysmography. We derive the NLCI as the normalized difference in SampEn between the original and surrogate data sets (Dhingra et al., 2011; Jacono et al., 2011; Young et al., 2019).

In critical illness (e.g., sepsis) breathing variability decreases (Askanazi et al., 1979; Warren et al., 2018). This could occur by changes in either the random or deterministic components of variability, or both. In our study, we evaluated how the deterministic component or NLCI changed following injections of LPS. Specifically, the NLCI in the LPS-treated group increased over time, indicating greater predictability (loss of variability) in the waveform pattern. In contrast, the NLCI for the PBS-treated group remained low, reflecting a maintenance of normal VPV in control rats. Consistent with an LPS-evoked increase in deterministic component in variability, mutual information (MI), a measurement of the probability of knowing the value of a future point based on the reference point, increased for the LPS-treated group and was greater than that for the PBS-treated group. Taken together, rats with inflammation had more predictable ventilatory pattern and thus a decrease in VPV.

### 4.3 Cardiorespiratory coupling (CRC) during systemic inflammation

Integrated network connectivity between physiologic systems such as respiratory and cardiovascular systems exhibit complex dynamics interactions. Different approaches examine phase transitions across physiological states including cardiorespiratory phase synchronization (CRPS) which manifests as a preference for heartbeats to occur at specific points relative to the phase of the respiratory cycle (Bartsch et al., 2014; Lucchini et al., 2018; Ren and Zhang, 2019). Another approach is time-delay stability (TDS), which examines the time delay between burst activation of one physiologic system compared to the following burst activation of the other system (Bartsch et al., 2015). Both CRPS and TDS measured decreased CRC in sleep transitions and aging. (Schumann et al., 2010; Bartsch et al., 2012; 2015). Another well-established CRC measurement is respiratory sinus arrhythmia (RSA), where HR increase during inspiration and decreases during expiration. This coordination of cardiovascular and respiratory rhythms is facilitated by the control of phase synchronization between nerve discharges through the neural cardiorespiratory circuits localized within the brainstem (Schulz et al., 1985; Schäfer et al., 1998). This coordination may be mediated by the cardio-respiratory control circuits in the ventrolateral medulla.

Cardioventilatory Coupling (CVC) is another form of cardio-respiratory coupling that refers to the tendency for the last heartbeat in expiration to occur at a preferred latency before inspiratory onset (Galletly and Larsen, 1998; Friedman et al., 2012; Barnett et al., 2021). In contrast to RSA, CVC represents the influence of the cardiovascular system on respiration. Respiratory neurons, especially expiratory neurons in the brainstem are modulated by blood pressure (Dick and Morris, 2004). This modulation depends on the carotid body afferents as well as an intact dorsolateral pons (Baekey et al., 2008). Indeed, modeling recorded brainstem single-neuron activity during transient blood pressure pulses supported baroreceptor feedback, which terminates in the nucleus tractus solitarii (nTS), excites post-inspiratory neurons, delays the onset of the next inspiration (Barnett et al., 2021).

During inflammation, pro-inflammatory cytokines, expressed in nTS decreased efficacy in neurotransmission between primary afferent inputs and the first order neurons in the nTS (Litvin et al., 2018; 2020; Getsy et al., 2019). Further, bilateral focal injections of pro-inflammatory cytokine IL-1β in the nTS ablates CVC (Hsieh et al., 2014).

Thus, to extend on our previous results of decreased CVC in an inflamed brainstem, we utilized two distinct forms of CRC (HRV and CVC) in this study with LPS rats. Here, HRV refers to the variability of HR in the frequency-domain. The respiratory modulation of HR relies on the expiratory modulation of vagal nerve activity (Laborde et al., 2017) while CVC refers to onset of inspiration at a preferred latency after the last heartbeat in expiration and it relies on baroreceptor feedback exciting post inspiratory neurons and delaying the inspiratory onset (Barnett et al., 2021). Thus, the neural substrate of HRV and CVC appears to be distinct. We report here that CVC decreases in LPS-treated rats. While the relative HF component of the PSD did not decrease in our study, others have reported a decrease in the HRV (Ahmad et al., 2009; Fairchild et al., 2009; Arnold et al., 2012; de Castilho et al., 2018). This difference could be due to magnitude of the dynamic of the neuro-inflammatory response.

### 4.4 Common factors influence cardiorespiratory Control during systemic inflammation

In our study, NLCI was correlated to fR. However, increases in NLCI could be dissociated from the increase in fR. NLCI increased prior to the increase in fR on Day 1 and the highest NLCI values occurred on Day 3 whereas peak fR on Day 2. Nevertheless, both NLCI and fR track progression of severity of inflammation. In a prior study evaluating NLCI as a potential biomarker in acute lung injury, in plots of receiver operating characteristic (ROC) curves, NLCI had a similar area (0.933) under the curve as fR (0.932) (Young et al., 2019). If increases in NLCI precede those of fR, then NLCI can be considered as an early index of neural inflammation and potentially as a noninvasive metric identifying the development of a dysregulated inflammatory response leading to sepsis.

We expected a negative correlation between CVC and NLCI given that potential brainstem inflammation associated with systemic inflammation reduces the efficacy primary sensory input. This is consistent with a decrease in respiratory pattern variability observed after blocking sensory input from the area of the carotid bifurcation (Dick et al., 2004; McMullan et al., 2009).

### 4.5 Limitations

An emphasis of this analysis was the repeatability of neighboring epochs, but the episodic nature of the epochs raises the issue of granularity, catching the progression. We analyzed multiple epochs (approximately 3 every hour). Continuous analysis of biologic rhythms will require automation of the analysis. In a prior study (Vandendriessche et al., 2017a), we published a generalizable framework that plotted multiscalar values on a state space that reflected 17 physiologically-related classes. The extremes were: 1) signals with high complexity dynamics and stable long-range correlations, which corresponded to “healthy” dynamics, and 2) signals, which were very random or very regular, both of which indicated pathophysiologic states underlying the dynamics. This framework allowed visual tracking of the patient’s state at the bedside.

Our study occurred between 08:00 and 16:00. Considering that rats are nocturnal (Bonmati-Carrion et al., 2017), a disruption in the sleep/wake cycle may be an important factor in these results. Numerous processes in the body have a circadian rhythm, from HR to hormone release (Chang et al., 2017). However, we recorded the rats during the same period of each day. Thus, even though we disrupted their circadian rhythm, we consider the stable HR observed with PBS injections as indicating that the results of the LPS-treated rats result from the LPS injections rather than the disruption of the sleep-wake cycle.

## 5 Conclusion

We can conclude that NLCI is a viable statistic and biometric in that it is reproducible during stable breathing and reflects changes in the pattern of breathing in response to repeated LPS-injections. Furthermore, while the response to LPS injections varied for the group, in particular several indices including HRV, HR, BP, fR, our analytical tools (CVC and NLCI) had consistent changes for a subset of rats. In particular, the increase in predictability of the respiratory pattern occurred in the LPS-treated rats.

## Data Availability

The original contributions presented in the study are included in the article/Supplementary Material, further inquiries can be directed to the corresponding author.
