# Supplementary material for: Dynamics of ventilatory pattern variability and Cardioventilatory Coupling during systemic inflammation in rats
Source: Front Netw Physiol. 2023 Jul 31;3:1038531. doi: 10.3389/fnetp.2023.1038531 (PMC10423997; doi:10.3389/fnetp.2023.1038531)

**Tables**

Table 1: Respiratory cycle duration statistics. Hours of the days which do not contain epoch data are represented by --. Epochs which only had one epoch do not contain standard deviation and are represented by *.


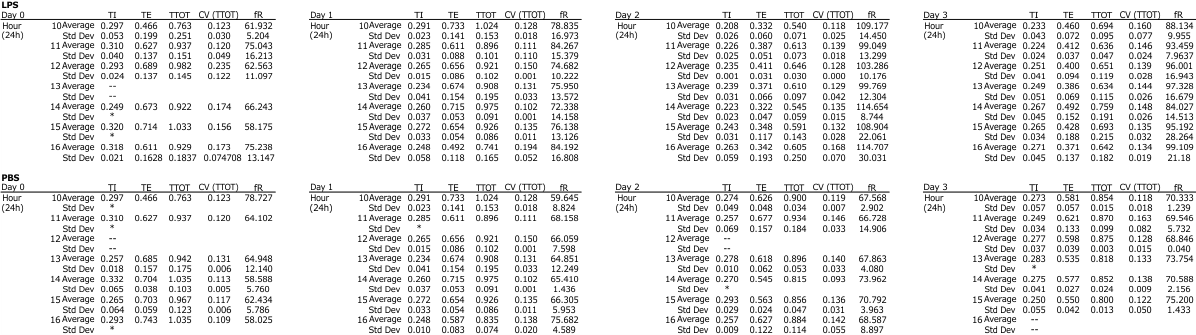


Table 2: Ventilatory pattern variability statistics. Hours of the days which do not contain epoch data are represented by --. Epochs which only had one epoch do not contain standard deviation and are represented by *.


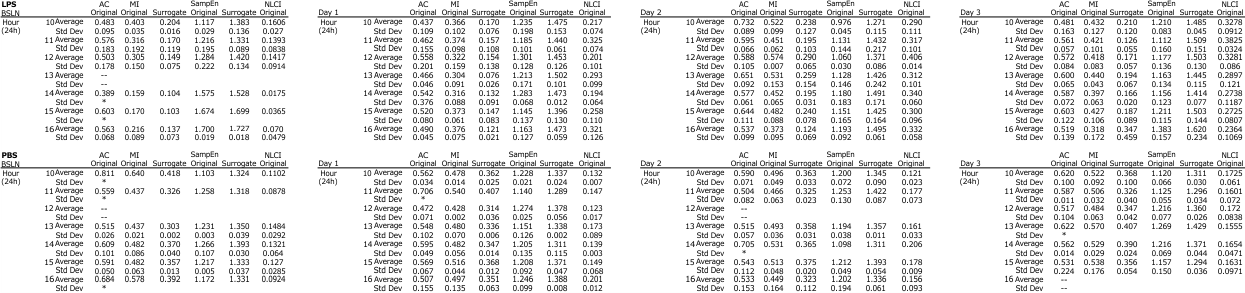


Table 3: CVC and HRV statistics. Hours of the days which do not contain epoch data are represented by --. Epochs which only had one epoch do not contain standard deviation and are represented by *.


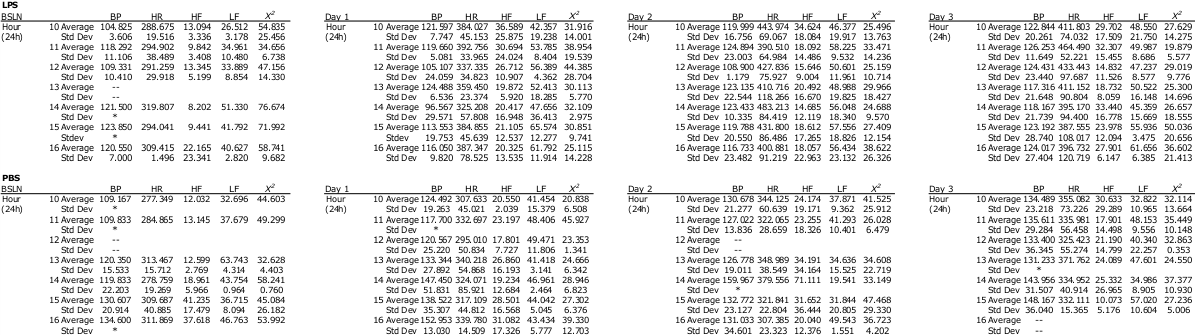

Supplement: Supplementary file 1 [file Table1.DOCX]
